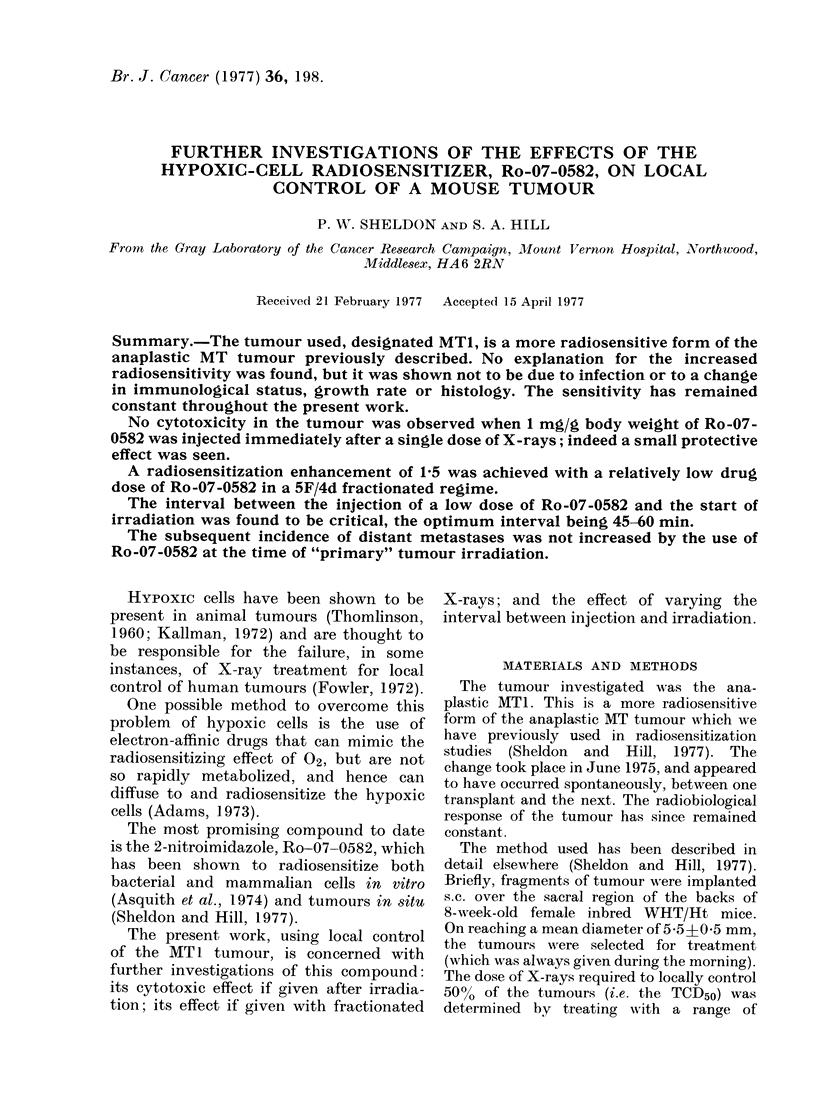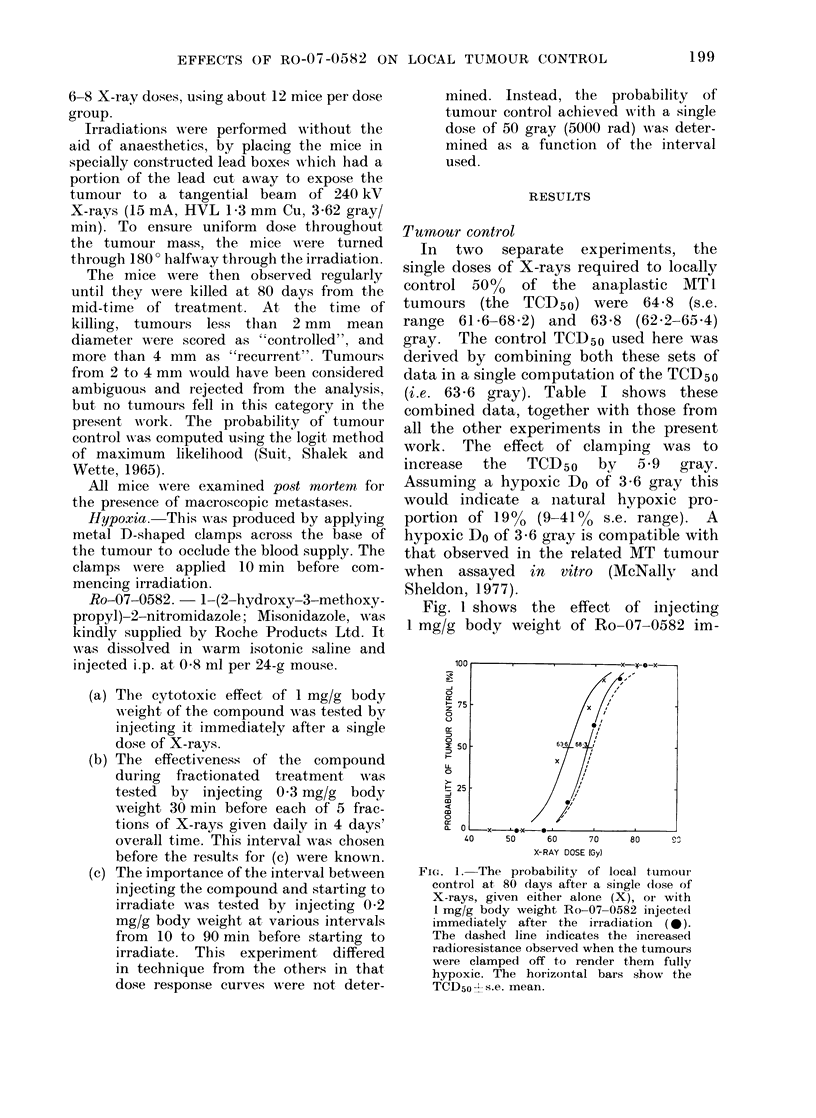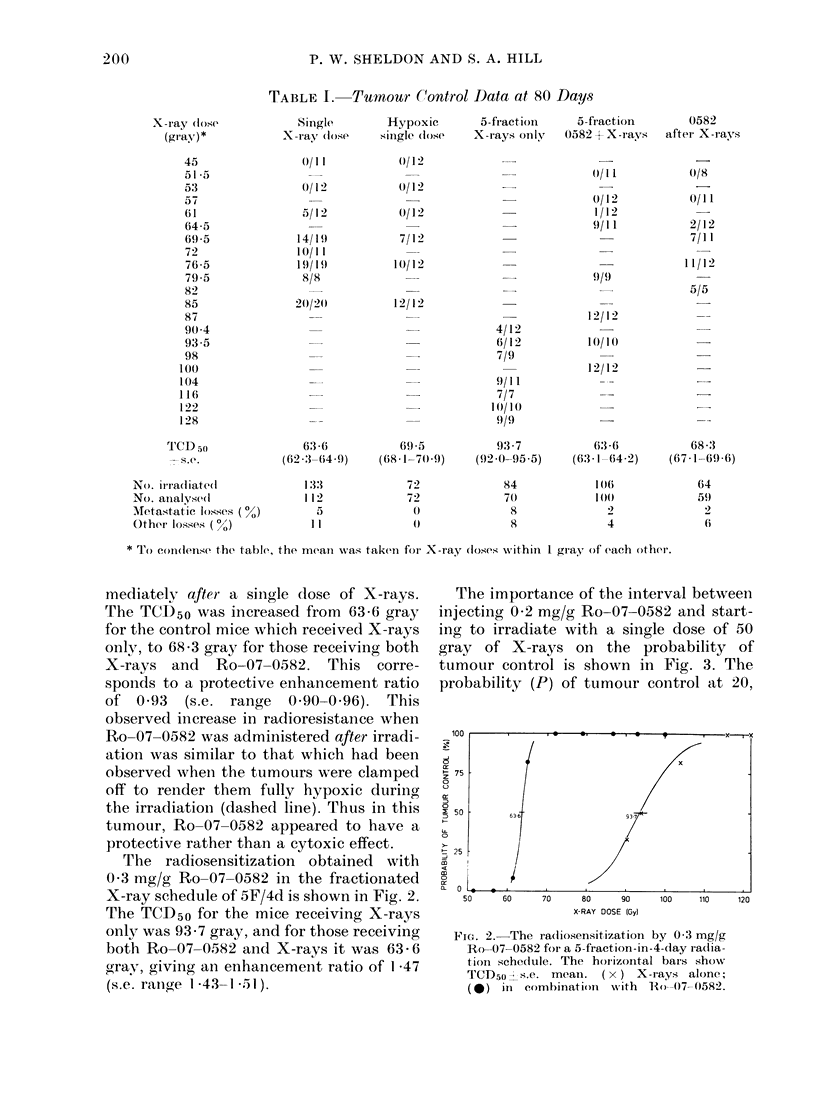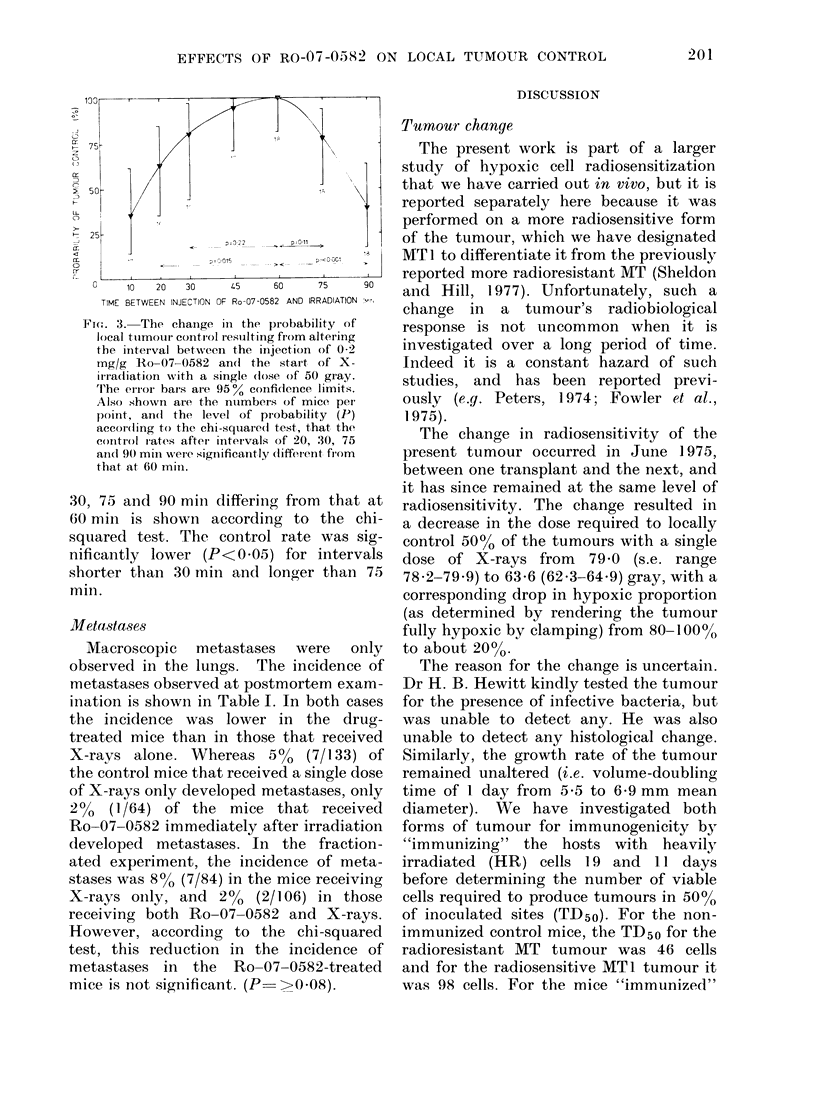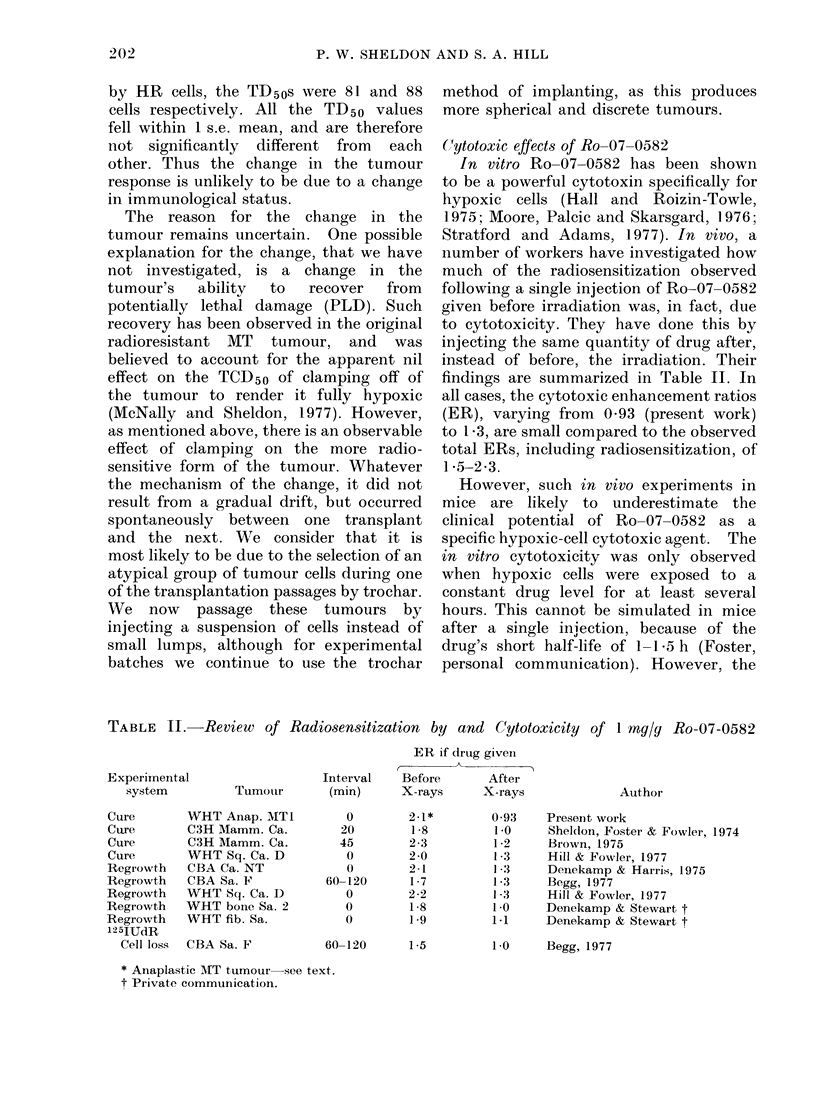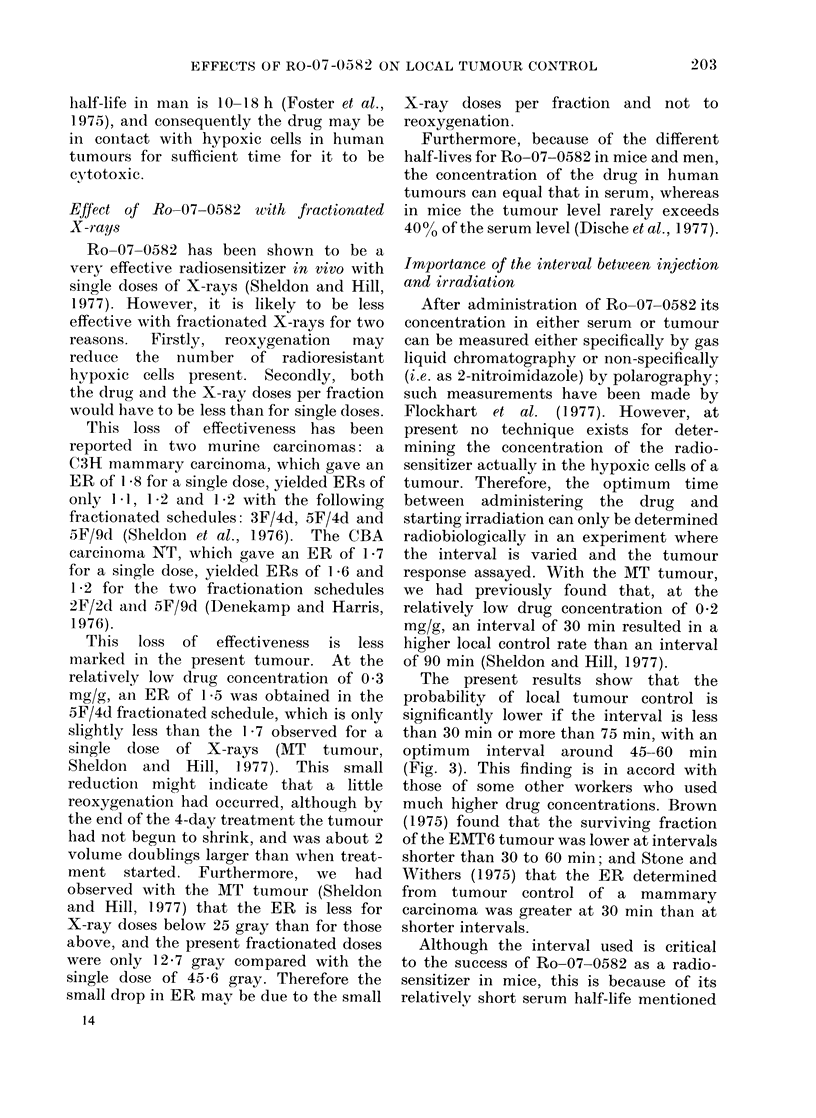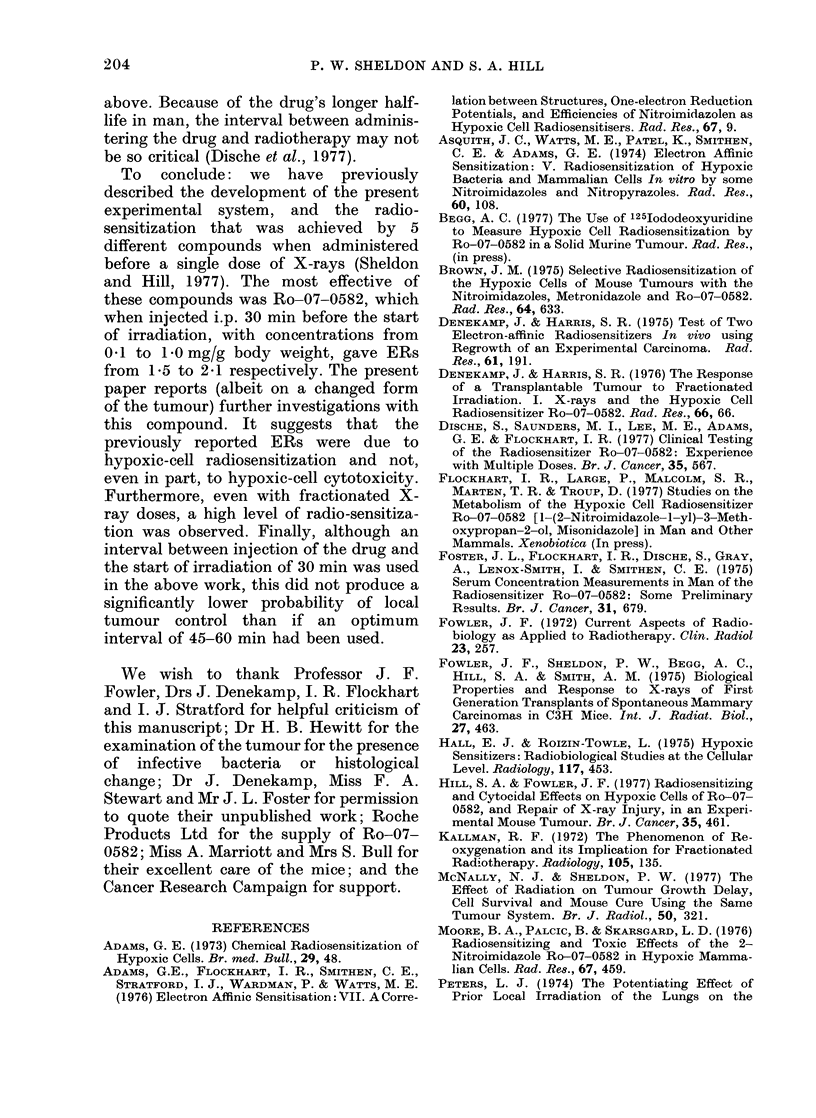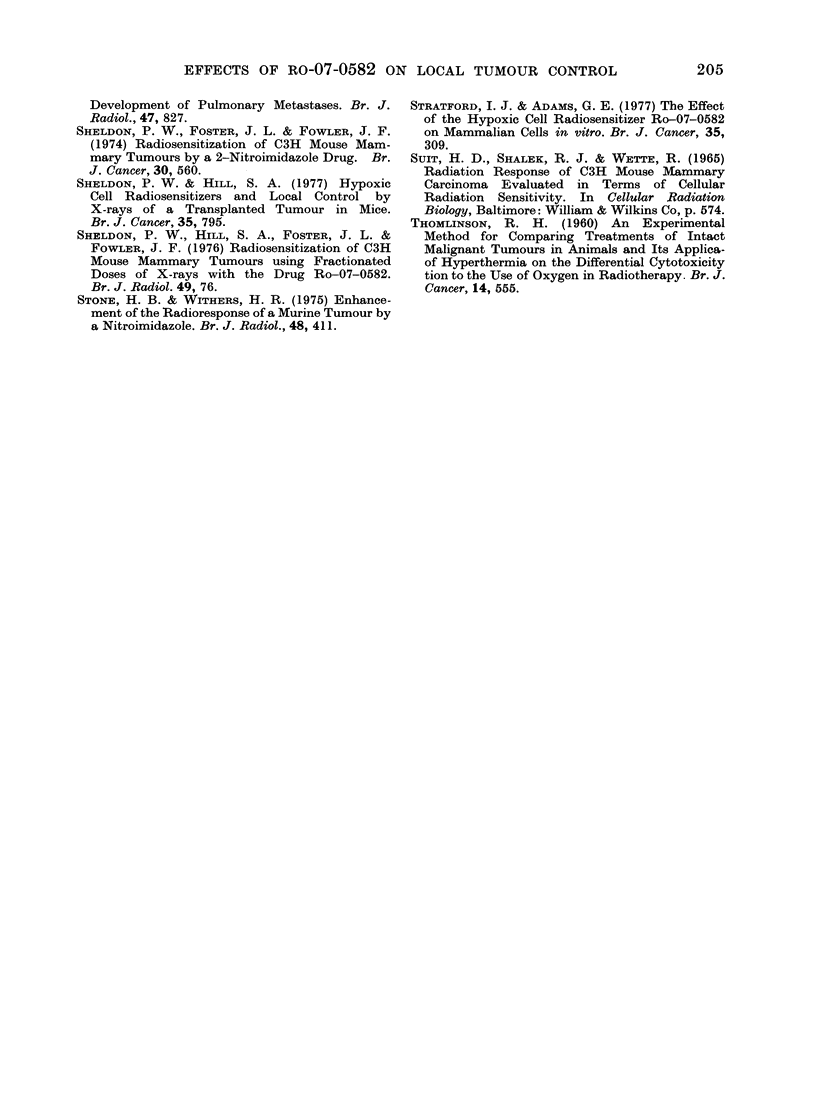# Further investigations of the effects of the hypoxic-cell radiosensitizer, Ro-07-0582, on local control of a mouse tumour.

**DOI:** 10.1038/bjc.1977.178

**Published:** 1977-08

**Authors:** P. W. Sheldon, S. A. Hill

## Abstract

The tumour used, designated MT1, is a more radiosensitive form of the anaplastic MT tumour previously described. No explanation for the increased radiosensitivity was found, but it was shown not to be due to infection or to a change in immunological status, growth rate or histology. The sensitivity has remained constant throughout the present work. No cytotoxicity in the tumour was observed when 1 mg/g body weight of Ro-07-0582 was injected immediately after a single dose of X-rays; indeed a small protective effect was seen. A radiosensitization enhancement of 1-5 was achieved with a relatively low drug dose of Ro-07-0582 in a 5F/4d fractionated regime. The interval between the injection of a low dose of Ro-07-0582 and the start of irradiation was found to be critical, the optimum interval being 45-60 min. The subsequent incidence of distant metastases was not increased by the use of Ro-07-0582 at the time of "primary" tumour irradiation.


*Br. J. Cancer* (1977) **36**, 198.

### FURTHER INVESTIGATIONS OF THE EFFECTS OF THE HYPOXIC-CELL RADIOSENSITIZER, Ro-07-0582, ON LOCAL CONTROL OF A MOUSE TUMOUR

P. W. SHELDON AND S. A. HILL

*From the Gray Laboratory of the Cancer Research Campaign, Mount Vernon Hospital, Northwood, Middlesex, HA6 2RN*

Received 21 February 1977 Accepted 15 April 1977

**Summary.—The tumour used, designated MT1, is a more radiosensitive form of the anaplastic MT tumour previously described. No explanation for the increased radiosensitivity was found, but it was shown not to be due to infection or to a change in immunological status, growth rate or histology. The sensitivity has remained constant throughout the present work.**

**No cytotoxicity in the tumour was observed when 1 mg/g body weight of Ro-07-0582 was injected immediately after a single dose of X-rays; indeed a small protective effect was seen.**

**A radiosensitization enhancement of 1·5 was achieved with a relatively low drug dose of Ro-07-0582 in a 5F/4d fractionated regime.**

**The interval between the injection of a low dose of Ro-07-0582 and the start of irradiation was found to be critical, the optimum interval being 45–60 min.**

**The subsequent incidence of distant metastases was not increased by the use of Ro-07-0582 at the time of "primary" tumour irradiation.**

HYPOXIC cells have been shown to be present in animal tumours (Thomlinson, 1960; Kallman, 1972) and are thought to be responsible for the failure, in some instances, of X-ray treatment for local control of human tumours (Fowler, 1972).

One possible method to overcome this problem of hypoxic cells is the use of electron-affinic drugs that can mimic the radiosensitizing effect of $O_2$, but are not so rapidly metabolized, and hence can diffuse to and radiosensitize the hypoxic cells (Adams, 1973).

The most promising compound to date is the 2-nitroimidazole, Ro–07–0582, which has been shown to radiosensitize both bacterial and mammalian cells *in vitro* (Asquith *et al.*, 1974) and tumours *in situ* (Sheldon and Hill, 1977).

The present work, using local control of the MT1 tumour, is concerned with further investigations of this compound: its cytotoxic effect if given after irradiation; its effect if given with fractionated X-rays; and the effect of varying the interval between injection and irradiation.

### MATERIALS AND METHODS

The tumour investigated was the anaplastic MT1. This is a more radiosensitive form of the anaplastic MT tumour which we have previously used in radiosensitization studies (Sheldon and Hill, 1977). The change took place in June 1975, and appeared to have occurred spontaneously, between one transplant and the next. The radiobiological response of the tumour has since remained constant.

The method used has been described in detail elsewhere (Sheldon and Hill, 1977). Briefly, fragments of tumour were implanted s.c. over the sacral region of the backs of 8-week-old female inbred WHT/Ht mice. On reaching a mean diameter of $5{\cdot}5 \pm 0{\cdot}5$ mm, the tumours were selected for treatment (which was always given during the morning). The dose of X-rays required to locally control 50% of the tumours (*i.e.* the $TCD_{50}$) was determined by treating with a range of

6–8 X-ray doses, using about 12 mice per dose group.

Irradiations were performed without the aid of anaesthetics, by placing the mice in specially constructed lead boxes which had a portion of the lead cut away to expose the tumour to a tangential beam of 240 kV X-rays (15 mA, HVL 1·3 mm Cu, 3·62 gray/min). To ensure uniform dose throughout the tumour mass, the mice were turned through 180° halfway through the irradiation.

The mice were then observed regularly until they were killed at 80 days from the mid-time of treatment. At the time of killing, tumours less than 2 mm mean diameter were scored as "controlled", and more than 4 mm as "recurrent". Tumours from 2 to 4 mm would have been considered ambiguous and rejected from the analysis, but no tumours fell in this category in the present work. The probability of tumour control was computed using the logit method of maximum likelihood (Suit, Shalek and Wette, 1965).

All mice were examined *post mortem* for the presence of macroscopic metastases.

*Hypoxia*.—This was produced by applying metal D-shaped clamps across the base of the tumour to occlude the blood supply. The clamps were applied 10 min before commencing irradiation.

*Ro*-07–0582. — 1–(2–hydroxy–3–methoxypropyl)–2–nitromidazole; Misonidazole, was kindly supplied by Roche Products Ltd. It was dissolved in warm isotonic saline and injected i.p. at 0·8 ml per 24-g mouse.

(a) The cytotoxic effect of 1 mg/g body weight of the compound was tested by injecting it immediately after a single dose of X-rays.
(b) The effectiveness of the compound during fractionated treatment was tested by injecting 0·3 mg/g body weight 30 min before each of 5 fractions of X-rays given daily in 4 days' overall time. This interval was chosen before the results for (c) were known.
(c) The importance of the interval between injecting the compound and starting to irradiate was tested by injecting 0·2 mg/g body weight at various intervals from 10 to 90 min before starting to irradiate. This experiment differed in technique from the others in that dose response curves were not determined. Instead, the probability of tumour control achieved with a single dose of 50 gray (5000 rad) was determined as a function of the interval used.

### RESULTS

### *Tumour control*

In two separate experiments, the single doses of X-rays required to locally control 50% of the anaplastic MT1 tumours (the $TCD_{50}$) were 64·8 (s.e. range 61·6–68·2) and 63·8 (62·2–65·4) gray. The control $TCD_{50}$ used here was derived by combining both these sets of data in a single computation of the $TCD_{50}$ (*i.e.* 63·6 gray). Table I shows these combined data, together with those from all the other experiments in the present work. The effect of clamping was to increase the $TCD_{50}$ by 5·9 gray. Assuming a hypoxic $D_0$ of 3·6 gray this would indicate a natural hypoxic proportion of 19% (9–41% s.e. range). A hypoxic $D_0$ of 3·6 gray is compatible with that observed in the related MT tumour when assayed *in vitro* (McNally and Sheldon, 1977).

Fig. 1 shows the effect of injecting 1 mg/g body weight of Ro–07–0582 im-

FIG. 1.—The probability of local tumour control at 80 days after a single dose of X-rays, given either alone (X), or with 1 mg/g body weight Ro–07–0582 injected immediately after the irradiation (●). The dashed line indicates the increased radioresistance observed when the tumours were clamped off to render them fully hypoxic. The horizontal bars show the $TCD_{50} \pm$ s.e. mean.

TABLE I.—*Tumour Control Data at 80 Days*

| X-ray dose (gray)* | Single X-ray dose | Hypoxic single dose | 5-fraction X-rays only | 5-fraction 0582 + X-rays | 0582 after X-rays |
|---|---|---|---|---|---|
| 45 | 0/11 | 0/12 | — | — | — |
| 51·5 | — | — | — | 0/11 | 0/8 |
| 53 | 0/12 | 0/12 | — | — | — |
| 57 | — | — | — | 0/12 | 0/11 |
| 61 | 5/12 | 0/12 | — | 1/12 | — |
| 64·5 | — | — | — | 9/11 | 2/12 |
| 69·5 | 14/19 | 7/12 | — | — | 7/11 |
| 72 | 10/11 | — | — | — | — |
| 76·5 | 19/19 | 10/12 | — | — | 11/12 |
| 79·5 | 8/8 | — | — | 9/9 | — |
| 82 | — | — | — | — | 5/5 |
| 85 | 20/20 | 12/12 | — | — | — |
| 87 | — | — | — | 12/12 | — |
| 90·4 | — | — | 4/12 | — | — |
| 93·5 | — | — | 6/12 | 10/10 | — |
| 98 | — | — | 7/9 | — | — |
| 100 | — | — | — | 12/12 | — |
| 104 | — | — | 9/11 | — | — |
| 116 | — | — | 7/7 | — | — |
| 122 | — | — | 10/10 | — | — |
| 128 | — | — | 9/9 | — | — |
| $TCD_{50}$ | 63·6 | 69·5 | 93·7 | 63·6 | 68·3 |
| ± s.e. | (62·3–64·9) | (68·1–70·9) | (92·0–95·5) | (63·1–64·2) | (67·1–69·6) |
| No. irradiated | 133 | 72 | 84 | 106 | 64 |
| No. analysed | 112 | 72 | 70 | 100 | 59 |
| Metastatic losses (%) | 5 | 0 | 8 | 2 | 2 |
| Other losses (%) | 11 | 0 | 8 | 4 | 6 |

* To condense the table, the mean was taken for X-ray doses within 1 gray of each other.

mediately *after* a single dose of X-rays. The $TCD_{50}$ was increased from 63·6 gray for the control mice which received X-rays only, to 68·3 gray for those receiving both X-rays and Ro–07–0582. This corresponds to a protective enhancement ratio of 0·93 (s.e. range 0·90–0·96). This observed increase in radioresistance when Ro–07–0582 was administered *after* irradiation was similar to that which had been observed when the tumours were clamped off to render them fully hypoxic during the irradiation (dashed line). Thus in this tumour, Ro–07–0582 appeared to have a protective rather than a cytoxic effect.

The radiosensitization obtained with 0·3 mg/g Ro–07–0582 in the fractionated X-ray schedule of 5F/4d is shown in Fig. 2. The $TCD_{50}$ for the mice receiving X-rays only was 93·7 gray, and for those receiving both Ro–07–0582 and X-rays it was 63·6 gray, giving an enhancement ratio of 1·47 (s.e. range 1·43–1·51).

The importance of the interval between injecting 0·2 mg/g Ro–07–0582 and starting to irradiate with a single dose of 50 gray of X-rays on the probability of tumour control is shown in Fig. 3. The probability ($P$) of tumour control at 20,

FIG. 2.—The radiosensitization by 0·3 mg/g Ro–07–0582 for a 5-fraction-in-4-day radiation schedule. The horizontal bars show $TCD_{50}$ ± s.e. mean. (×) X-rays alone; (●) in combination with Ro–07–0582.

FIG. 3.—The change in the probability of local tumour control resulting from altering the interval between the injection of 0·2 mg/g Ro-07-0582 and the start of X-irradiation with a single dose of 50 gray. The error bars are 95% confidence limits. Also shown are the numbers of mice per point, and the level of probability ($P$) according to the chi-squared test, that the control rates after intervals of 20, 30, 75 and 90 min were significantly different from that at 60 min.

30, 75 and 90 min differing from that at 60 min is shown according to the chi-squared test. The control rate was significantly lower ($P<0·05$) for intervals shorter than 30 min and longer than 75 min.

*Metastases*

Macroscopic metastases were only observed in the lungs. The incidence of metastases observed at postmortem examination is shown in Table I. In both cases the incidence was lower in the drug-treated mice than in those that received X-rays alone. Whereas 5% (7/133) of the control mice that received a single dose of X-rays only developed metastases, only 2% (1/64) of the mice that received Ro–07–0582 immediately after irradiation developed metastases. In the fractionated experiment, the incidence of metastases was 8% (7/84) in the mice receiving X-rays only, and 2% (2/106) in those receiving both Ro–07–0582 and X-rays. However, according to the chi-squared test, this reduction in the incidence of metastases in the Ro–07–0582-treated mice is not significant. ($P= \geq 0·08$).

### DISCUSSION

*Tumour change*

The present work is part of a larger study of hypoxic cell radiosensitization that we have carried out *in vivo*, but it is reported separately here because it was performed on a more radiosensitive form of the tumour, which we have designated MT1 to differentiate it from the previously reported more radioresistant MT (Sheldon and Hill, 1977). Unfortunately, such a change in a tumour's radiobiological response is not uncommon when it is investigated over a long period of time. Indeed it is a constant hazard of such studies, and has been reported previously (*e.g.* Peters, 1974; Fowler *et al.*, 1975).

The change in radiosensitivity of the present tumour occurred in June 1975, between one transplant and the next, and it has since remained at the same level of radiosensitivity. The change resulted in a decrease in the dose required to locally control 50% of the tumours with a single dose of X-rays from 79·0 (s.e. range 78·2–79·9) to 63·6 (62·3–64·9) gray, with a corresponding drop in hypoxic proportion (as determined by rendering the tumour fully hypoxic by clamping) from 80–100% to about 20%.

The reason for the change is uncertain. Dr H. B. Hewitt kindly tested the tumour for the presence of infective bacteria, but was unable to detect any. He was also unable to detect any histological change. Similarly, the growth rate of the tumour remained unaltered (*i.e.* volume-doubling time of 1 day from 5·5 to 6·9 mm mean diameter). We have investigated both forms of tumour for immunogenicity by "immunizing" the hosts with heavily irradiated (HR) cells 19 and 11 days before determining the number of viable cells required to produce tumours in 50% of inoculated sites ($TD_{50}$). For the non-immunized control mice, the $TD_{50}$ for the radioresistant MT tumour was 46 cells and for the radiosensitive MT1 tumour it was 98 cells. For the mice "immunized"

by HR cells, the $TD_{50}$s were 81 and 88 cells respectively. All the $TD_{50}$ values fell within 1 s.e. mean, and are therefore not significantly different from each other. Thus the change in the tumour response is unlikely to be due to a change in immunological status.

The reason for the change in the tumour remains uncertain. One possible explanation for the change, that we have not investigated, is a change in the tumour's ability to recover from potentially lethal damage (PLD). Such recovery has been observed in the original radioresistant MT tumour, and was believed to account for the apparent nil effect on the $TCD_{50}$ of clamping off of the tumour to render it fully hypoxic (McNally and Sheldon, 1977). However, as mentioned above, there is an observable effect of clamping on the more radiosensitive form of the tumour. Whatever the mechanism of the change, it did not result from a gradual drift, but occurred spontaneously between one transplant and the next. We consider that it is most likely to be due to the selection of an atypical group of tumour cells during one of the transplantation passages by trochar. We now passage these tumours by injecting a suspension of cells instead of small lumps, although for experimental batches we continue to use the trochar method of implanting, as this produces more spherical and discrete tumours.

*Cytotoxic effects of Ro*–07–0582

*In vitro* Ro–07–0582 has been shown to be a powerful cytotoxin specifically for hypoxic cells (Hall and Roizin-Towle, 1975; Moore, Palcic and Skarsgard, 1976; Stratford and Adams, 1977). *In vivo,* a number of workers have investigated how much of the radiosensitization observed following a single injection of Ro–07–0582 given before irradiation was, in fact, due to cytotoxicity. They have done this by injecting the same quantity of drug after, instead of before, the irradiation. Their findings are summarized in Table II. In all cases, the cytotoxic enhancement ratios (ER), varying from 0·93 (present work) to 1·3, are small compared to the observed total ERs, including radiosensitization, of 1·5–2·3.

However, such *in vivo* experiments in mice are likely to underestimate the clinical potential of Ro–07–0582 as a specific hypoxic-cell cytotoxic agent. The *in vitro* cytotoxicity was only observed when hypoxic cells were exposed to a constant drug level for at least several hours. This cannot be simulated in mice after a single injection, because of the drug's short half-life of 1–1·5 h (Foster, personal communication). However, the

TABLE II.—*Review of Radiosensitization by and Cytotoxicity of* 1 *mg/g Ro*-07-0582

| Experimental system | Tumour | Interval (min) | ER if drug given: Before X-rays | After X-rays | Author |
|---|---|---|---|---|---|
| Cure | WHT Anap. MT1 | 0 | 2·1* | 0·93 | Present work |
| Cure | C3H Mamm. Ca. | 20 | 1·8 | 1·0 | Sheldon, Foster & Fowler, 1974 |
| Cure | C3H Mamm. Ca. | 45 | 2·3 | 1·2 | Brown, 1975 |
| Cure | WHT Sq. Ca. D | 0 | 2·0 | 1·3 | Hill & Fowler, 1977 |
| Regrowth | CBA Ca. NT | 0 | 2·1 | 1·3 | Denekamp & Harris, 1975 |
| Regrowth | CBA Sa. F | 60–120 | 1·7 | 1·3 | Begg, 1977 |
| Regrowth | WHT Sq. Ca. D | 0 | 2·2 | 1·3 | Hill & Fowler, 1977 |
| Regrowth | WHT bone Sa. 2 | 0 | 1·8 | 1·0 | Denekamp & Stewart † |
| Regrowth | WHT fib. Sa. | 0 | 1·9 | 1·1 | Denekamp & Stewart † |
| $^{125}$IUdR Cell loss | CBA Sa. F | 60–120 | 1·5 | 1·0 | Begg, 1977 |

* Anaplastic MT tumour—see text.
† Private communication.

half-life in man is 10–18 h (Foster *et al.*, 1975), and consequently the drug may be in contact with hypoxic cells in human tumours for sufficient time for it to be cytotoxic.

*Effect of Ro–07–0582 with fractionated X-rays*

Ro–07–0582 has been shown to be a very effective radiosensitizer *in vivo* with single doses of X-rays (Sheldon and Hill, 1977). However, it is likely to be less effective with fractionated X-rays for two reasons. Firstly, reoxygenation may reduce the number of radioresistant hypoxic cells present. Secondly, both the drug and the X-ray doses per fraction would have to be less than for single doses.

This loss of effectiveness has been reported in two murine carcinomas: a C3H mammary carcinoma, which gave an ER of 1·8 for a single dose, yielded ERs of only 1·1, 1·2 and 1·2 with the following fractionated schedules: 3F/4d, 5F/4d and 5F/9d (Sheldon *et al.*, 1976). The CBA carcinoma NT, which gave an ER of 1·7 for a single dose, yielded ERs of 1·6 and 1·2 for the two fractionation schedules 2F/2d and 5F/9d (Denekamp and Harris, 1976).

This loss of effectiveness is less marked in the present tumour. At the relatively low drug concentration of 0·3 mg/g, an ER of 1·5 was obtained in the 5F/4d fractionated schedule, which is only slightly less than the 1·7 observed for a single dose of X-rays (MT tumour, Sheldon and Hill, 1977). This small reduction might indicate that a little reoxygenation had occurred, although by the end of the 4-day treatment the tumour had not begun to shrink, and was about 2 volume doublings larger than when treatment started. Furthermore, we had observed with the MT tumour (Sheldon and Hill, 1977) that the ER is less for X-ray doses below 25 gray than for those above, and the present fractionated doses were only 12·7 gray compared with the single dose of 45·6 gray. Therefore the small drop in ER may be due to the small X-ray doses per fraction and not to reoxygenation.

Furthermore, because of the different half-lives for Ro–07–0582 in mice and men, the concentration of the drug in human tumours can equal that in serum, whereas in mice the tumour level rarely exceeds 40% of the serum level (Dische *et al.*, 1977).

*Importance of the interval between injection and irradiation*

After administration of Ro–07–0582 its concentration in either serum or tumour can be measured either specifically by gas liquid chromatography or non-specifically (*i.e.* as 2-nitroimidazole) by polarography; such measurements have been made by Flockhart *et al.* (1977). However, at present no technique exists for determining the concentration of the radiosensitizer actually in the hypoxic cells of a tumour. Therefore, the optimum time between administering the drug and starting irradiation can only be determined radiobiologically in an experiment where the interval is varied and the tumour response assayed. With the MT tumour, we had previously found that, at the relatively low drug concentration of 0·2 mg/g, an interval of 30 min resulted in a higher local control rate than an interval of 90 min (Sheldon and Hill, 1977).

The present results show that the probability of local tumour control is significantly lower if the interval is less than 30 min or more than 75 min, with an optimum interval around 45–60 min (Fig. 3). This finding is in accord with those of some other workers who used much higher drug concentrations. Brown (1975) found that the surviving fraction of the EMT6 tumour was lower at intervals shorter than 30 to 60 min; and Stone and Withers (1975) that the ER determined from tumour control of a mammary carcinoma was greater at 30 min than at shorter intervals.

Although the interval used is critical to the success of Ro–07–0582 as a radiosensitizer in mice, this is because of its relatively short serum half-life mentioned above. Because of the drug's longer half-life in man, the interval between administering the drug and radiotherapy may not be so critical (Dische *et al.*, 1977).

To conclude: we have previously described the development of the present experimental system, and the radiosensitization that was achieved by 5 different compounds when administered before a single dose of X-rays (Sheldon and Hill, 1977). The most effective of these compounds was Ro–07–0582, which when injected i.p. 30 min before the start of irradiation, with concentrations from 0·1 to 1·0 mg/g body weight, gave ERs from 1·5 to 2·1 respectively. The present paper reports (albeit on a changed form of the tumour) further investigations with this compound. It suggests that the previously reported ERs were due to hypoxic-cell radiosensitization and not, even in part, to hypoxic-cell cytotoxicity. Furthermore, even with fractionated X-ray doses, a high level of radio-sensitization was observed. Finally, although an interval between injection of the drug and the start of irradiation of 30 min was used in the above work, this did not produce a significantly lower probability of local tumour control than if an optimum interval of 45–60 min had been used.

We wish to thank Professor J. F. Fowler, Drs J. Denekamp, I. R. Flockhart and I. J. Stratford for helpful criticism of this manuscript; Dr H. B. Hewitt for the examination of the tumour for the presence of infective bacteria or histological change; Dr J. Denekamp, Miss F. A. Stewart and Mr J. L. Foster for permission to quote their unpublished work; Roche Products Ltd for the supply of Ro–07–0582; Miss A. Marriott and Mrs S. Bull for their excellent care of the mice; and the Cancer Research Campaign for support.

### REFERENCES

Adams, G. E. (1973) Chemical Radiosensitization of Hypoxic Cells. *Br. med. Bull.*, **29**, 48.

Adams, G.E., Flockhart, I. R., Smithen, C. E., Stratford, I. J., Wardman, P. & Watts, M. E. (1976) Electron Affinic Sensitisation: VII. A Correlation between Structures, One-electron Reduction Potentials, and Efficiencies of Nitroimidazolen as Hypoxic Cell Radiosensitisers. *Rad. Res.*, **67**, 9.

Asquith, J. C., Watts, M. E., Patel, K., Smithen, C. E. & Adams, G. E. (1974) Electron Affinic Sensitization: V. Radiosensitization of Hypoxic Bacteria and Mammalian Cells *In vitro* by some Nitroimidazoles and Nitropyrazoles. *Rad. Res.*, **60**, 108.

Begg, A. C. (1977) The Use of $^{125}$Iododeoxyuridine to Measure Hypoxic Cell Radiosensitization by Ro–07–0582 in a Solid Murine Tumour. *Rad. Res.*, (in press).

Brown, J. M. (1975) Selective Radiosensitization of the Hypoxic Cells of Mouse Tumours with the Nitroimidazoles, Metronidazole and Ro–07–0582. *Rad. Res.*, **64**, 633.

Denekamp, J. & Harris, S. R. (1975) Test of Two Electron-affinic Radiosensitizers *In vivo* using Regrowth of an Experimental Carcinoma. *Rad. Res.*, **61**, 191.

Denekamp, J. & Harris, S. R. (1976) The Response of a Transplantable Tumour to Fractionated Irradiation. I. X-rays and the Hypoxic Cell Radiosensitizer Ro–07–0582. *Rad. Res.*, **66**, 66.

Dische, S., Saunders, M. I., Lee, M. E., Adams, G. E. & Flockhart, I. R. (1977) Clinical Testing of the Radiosensitizer Ro–07–0582: Experience with Multiple Doses. *Br. J. Cancer*, **35**, 567.

Flockhart, I. R., Large, P., Malcolm, S. R., Marten, T. R. & Troup, D. (1977) Studies on the Metabolism of the Hypoxic Cell Radiosensitizer Ro–07–0582 [1–(2–Nitroimidazole–1–yl)–3–Methoxypropan–2–ol, Misonidazole] in Man and Other Mammals. *Xenobiotica* (In press).

Foster, J. L., Flockhart, I. R., Dische, S., Gray, A., Lenox-Smith, I. & Smithen, C. E. (1975) Serum Concentration Measurements in Man of the Radiosensitizer Ro–07–0582: Some Preliminary Results. *Br. J. Cancer*, **31**, 679.

Fowler, J. F. (1972) Current Aspects of Radiobiology as Applied to Radiotherapy. *Clin. Radiol* **23**, 257.

Fowler, J. F., Sheldon, P. W., Begg, A. C., Hill, S. A. & Smith, A. M. (1975) Biological Properties and Response to X-rays of First Generation Transplants of Spontaneous Mammary Carcinomas in C3H Mice. *Int. J. Radiat. Biol.*, **27**, 463.

Hall, E. J. & Roizin-Towle, L. (1975) Hypoxic Sensitizers: Radiobiological Studies at the Cellular Level. *Radiology*, **117**, 453.

Hill, S. A. & Fowler, J. F. (1977) Radiosensitizing and Cytocidal Effects on Hypoxic Cells of Ro–07–0582, and Repair of X-ray Injury, in an Experimental Mouse Tumour. *Br. J. Cancer*, **35**, 461.

Kallman, R. F. (1972) The Phenomenon of Reoxygenation and its Implication for Fractionated Radiotherapy. *Radiology*, **105**, 135.

McNally, N. J. & Sheldon, P. W. (1977) The Effect of Radiation on Tumour Growth Delay, Cell Survival and Mouse Cure Using the Same Tumour System. *Br. J. Radiol.*, **50**, 321.

Moore, B. A., Palcic, B. & Skarsgard, L. D. (1976) Radiosensitizing and Toxic Effects of the 2–Nitroimidazole Ro–07–0582 in Hypoxic Mammalian Cells. *Rad. Res.*, **67**, 459.

Peters, L. J. (1974) The Potentiating Effect of Prior Local Irradiation of the Lungs on the

Development of Pulmonary Metastases. *Br. J. Radiol.*, **47**, 827.

SHELDON, P. W., FOSTER, J. L. & FOWLER, J. F. (1974) Radiosensitization of C3H Mouse Mammary Tumours by a 2–Nitroimidazole Drug. *Br. J. Cancer*, **30**, 560.

SHELDON, P. W. & HILL, S. A. (1977) Hypoxic Cell Radiosensitizers and Local Control by X-rays of a Transplanted Tumour in Mice. *Br. J. Cancer*, **35**, 795.

SHELDON, P. W., HILL, S. A., FOSTER, J. L. & FOWLER, J. F. (1976) Radiosensitization of C3H Mouse Mammary Tumours using Fractionated Doses of X-rays with the Drug Ro–07–0582. *Br. J. Radiol.* **49**, 76.

STONE, H. B. & WITHERS, H. R. (1975) Enhancement of the Radioresponse of a Murine Tumour by a Nitroimidazole. *Br. J. Radiol.*, **48**, 411.

STRATFORD, I. J. & ADAMS, G. E. (1977) The Effect of the Hypoxic Cell Radiosensitizer Ro–07–0582 on Mammalian Cells *in vitro*. *Br. J. Cancer*, **35**, 309.

SUIT, H. D., SHALEK, R. J. & WETTE, R. (1965) Radiation Response of C3H Mouse Mammary Carcinoma Evaluated in Terms of Cellular Radiation Sensitivity. In *Cellular Radiation Biology*, Baltimore: William & Wilkins Co, p. 574.

THOMLINSON, R. H. (1960) An Experimental Method for Comparing Treatments of Intact Malignant Tumours in Animals and Its Applica- of Hyperthermia on the Differential Cytotoxicity tion to the Use of Oxygen in Radiotherapy. *Br. J. Cancer*, **14**, 555.